# Sensory Evaluation through RATA and Sorting Task of Commercial and Traditional Panettones Sold in Peru

**DOI:** 10.3390/foods13101508

**Published:** 2024-05-13

**Authors:** Reynaldo J. Silva-Paz, Robert W. Ocrospoma-Dueñas, Yolanda M. Eguilas-Caushi, Rossy A. Padilla-Fabian, Nicodemo C. Jamanca-Gonzales

**Affiliations:** Escuela de Ingeniería en Industrias Alimentarias, Departamento de Ingeniería, Universidad Nacional de Barranca, Av. Toribio de Luzuriaga N° 376 Mz J. Urb. La Florida, Barranca 15169, Peruyeguilasc@unab.edu.pe (Y.M.E.-C.); rpadillaf@unab.edu.pe (R.A.P.-F.); njamanca@unab.edu.pe (N.C.J.-G.)

**Keywords:** panettone, consumer, discriminative, descriptive, acceptability

## Abstract

In Peru, the consumption of panettone has increased, highlighting the importance of its sensory aspect, quality and price for its acceptance. This study evaluated sensory, physicochemical, texture and color attributes in commercial and traditional panettones. The RATA descriptive test and the discriminative sorting task were used, with 168 and 92 consumers, respectively. In addition, acceptability and purchase intention were evaluated. Significant differences were found between the samples; the traditional panettone showed lower weight, pH and fat content. Regarding the color of the crust and crumb, differences were also observed between both types. Regarding texture, traditional panettone showed less hardness and chewiness compared to commercial ones. The sorting method allowed us to differentiate the samples, where consumers differentiated the traditional panettone from the commercial ones, although within the commercial ones, they also found differences. The RATA test showed a similar behavior, traditional panettones were described as spongy, with fruits and a strong smell, unlike the commercial ones characterized as greasy, brown and fibrous. It is concluded that sensory methods are useful to understand the quality of panettone along with the physicochemical parameters, which influence consumer preferences according to the sensory characteristics and the quality of the ingredients.

## 1. Introduction

Panettone, originally from Italy, has gained wide popularity in Peru [1]. This sweet bread has the appearance of a spongy, cylinder-shaped bun and is distinguished by its diverse content, which includes fortified flours, substitute flours from Andean grains, candied fruits, dried products, essences and flavorings. These ingredients give it sensory properties highly appreciated by consumers. The formulation of panettone can vary considerably, from the use of select inputs that include preferments, sourdough [2], to the use of premixes that significantly reduce production costs. This diversity is observed in the Peruvian market, where there are various brands of panettones, classified as commercial panettones (belonging to products recognized for their large volumes of production and sales) and traditional panettones, which have limited production (special formulations for customers with particular needs of quality and price).

Currently, new food trends demand natural, ecological products with properties to improve health [3]. Satisfying the specific needs of the consumer promotes innovation, using bioactive compounds [4], with low fat content [5], flour or flakes of Andean grains [6], even tubers such as orange-fleshed sweet potatoes [2]. The purchase of traditional products, as well as organic ones, is closely tied to consumer behavior linked to the place and quantity of purchase, motivated by optimization reasons, production planning, ecological interests, rural development and value chain considerations [7]. These factors are closely related to their quality, price, appearance, brand, availability and place of origin [8]. In Peru, there is a variety of commercial panettone brands produced with original recipes that cover a significant segment of the market. However, there is a trend towards healthier products, incorporating additional ingredients such as pecans, almonds or dried cranberries [9], the use of various types of flour (lucuma, maca, purple corn) and a greater diversity of complementary ingredients [10]. This trend creates an interesting opportunity for the development of traditional products, which typically display higher prices, akin to organic foods. This similarity poses an obstacle to the growth of their demand [11] for segments with specific sensory needs, whose recipes and sales are available on the social media platforms of the Peruvian market.

Understanding the consumer perception of a food product turns out to be a complex task that integrates intrinsic (sensory attributes) and extrinsic (packaging, brand, price and others) expectations of the product, as well as socioeconomic and ethical aspects [12]. Great importance is given to the nutritional content of foods, whose detailed information on labels is subject to regulations by the competent health authorities. Therefore, sensory evaluation focuses on establishing a connection with the consumer’s behavior, closely linked to their emotions when evaluating them [13]. Currently, there is an inclination towards the creation and innovation of food products, with the aim of achieving a balance between their nutritional and sensory properties.

Sensory evaluation as a tool not only allows for the level of the acceptance of foods but also quantifies them, through descriptive methods with consumers. Descriptive sensory analyses are distinguished from other sensory testing methods because they seek to profile a product based on all of its perceived sensory characteristics [14]. To date, there are many descriptive methods with consumers, used because they allow for reducing costs and training times, thus these methods can be classified into three families (i) methods based on the use of verbal terms (Flash Profile and CATA), (ii) methods similarity-based (free classification task and projective mapping also known as Napping^®^) and (iii) reference-based methods (polarized sensory positioning and pivot profile) [15]. There are references to CATA (Check-All-That-Apply) and its variations: RATA (Rate-All-That-Apply), TCATA (Temporal Check-All-That-Apply), TCATA fading (Temporal Check-All-That-Apply Fading) and CATA-I (Optimal Check-All-That-Apply) [16], Comparison of Free Multiple Sorting, Partial Napping, Napping, Flash Profiling and conventional profiling [17], projective mapping [18] and sorting task, all of which are used in the food industry to develop new products to offer the faster innovations that consumers expect [19] and include consumer insights [20], Flash Profile [21].

Descriptive methods such as CATA have been used in some baking products such as cookies with flour from tubers, fruits or cereals other than wheat [22], in sprouted wheat drinks [23], in sourdough bread made with a coconut water kefir starter [24], and in sorghum and cowpea cookies [25]; some trials have even been carried out incorporating the time to the Time Intensity analysis [26], the Flash Profile in porridge [15], and Free Sorting, which were applied in the evaluation of the impact of dry sourdough as an ingredient on the smell of the bread [27], or the sensory profile of corn tortillas [28], but there is still limited information on the application of sensory descriptive tests with consumers of panettone, with only some existing such as the application of CATA in panettone made with preferments, sourdough and commercial yeasts [29] or CATA in panettones processed with preferments [30]. This study aimed to evaluate the sensory attributes of four commercial and two traditional panettones most consumed in the Peruvian market through RATA (Rate-All-That-Apply) and sorting task tests, complemented with physicochemical, textural and colorimetric tests.

## 2. Materials and Methods

### 2.1. Sample of Panettone

Six samples of panettone were considered for the present study, which were selected by convenience. Four commercial brands were considered, with T1 and T2 being the two most recognized brands in the Peruvian market and T3 and T4 identified as intermediate quality brands. In addition, two traditional panettones, T5 and T6, were included. The selection of these samples was based on the cost of the product, and they were obtained in the central market of Barranca, Peru. Table 1 presents the characteristics of the panettone studied, describing their nutritional information, and a list of ingredients used in the production process.

### 2.2. Physicochemical Analysis

The physicochemical analyses of the panettone were as follows: moisture content using a stove (Binder brand, model FD53, Tuttlingen, Germany) [31], ash using a muffle oven (Thermo Concept brand, model KL15/11, Bremen, Germany) [32], pH with a potentiometer (Hanna HI320 brand, Woonsocket, RI, USA), acidity by gravimetry [33] in titration equipment (Titronic brand, model 500, Madrid, Spain) and fat using a semi-automatic fat extractor (Velp Scientifica brand, model SER 148, Usmate Velate, Italy) according to the Official AOAC methods [34]. Its diameter (cm) was determined using a digital electronic caliper-type Vernier caliper (Control Company Traceable brand, model SR44, Mexico City, Mexico), with a scale of 0–25 mm; weight was measured using an analytical balance (Sartorius brand, model Entris 224-1S, Gottingen, Germany).

### 2.3. Taking and Analyzing Images

To obtain images, cross sections were made in the samples. The shots were taken at a height of 40 cm from the sample using Motog (g) plus cellular equipment with f/1.8 1/40 5.53 mm (Motorola brand, model XT 2087-2, Schaumburg, IL, USA). The shape analysis was carried out from the images; for this, the brightness, contrast and threshold were adjusted and converted using the ImageJ^®^ Software version 1.53K (https://imagej.nih.gov/ij/ (accessed 8 January 2024)) [35]. To cut out the shapes, the online software Remove BG was used, available at the following link https://pixlr.com/es/remove-background/ (accessed 8 January 2024).

### 2.4. Instrumental Texture Test

Texture profile analysis (TPA) parameters were determined using a Brookfield Metek Texturometer model CTX, from the USA, using TexturePro V1.0 Build 19 software from Brookfield Engineering Labs Inc. The test conditions were as follows: TA25/1000 compression plate probe, double compression, speed 0.5 mm/s, with 4.0 mm penetration, 5 kg load cell, evaluating hardness, cohesiveness, chewiness and resilience [36]. The samples were sliced with a diameter of 45 mm and a height of 45 mm (cylindrical shape) extracted from the central part (crumb), and the measurements were made as soon as possible to avoid moisture loss. Texture parameters were measured in quadruplicate replicates.

### 2.5. Crumb and Crust Color Test

The color parameters of the crumb and crust were determined using a Colorimeter (PCE Instruments brand, model CSM 3, Tobarra, Spain), with an observation angle of 10° and Illuminant D65 at 420 nm. Color registration was carried out using the CIE-L* a* b* system. In this system, L* represents the luminosity, a* indicates the hue on the green (−)-to-red (+) axis and b* indicates the hue on the blue (−)-to-yellow (+) axis [30] and CIE-L*c*h*, where L denotes luminosity, c denotes chroma or saturation and h denotes the hue or hue angle [37].

### 2.6. Sensory Analysis

A study was carried out with 168 regular panettone consumers (51% women and 49% men), aged between 18 and 50 years, who evaluated the sensory characteristics of the product using the RATA (Rate-All-That-Apply) questions, with a 5-point rating scale. For this purpose, non-probabilistic sampling was used for convenience. Fifteen sensory terms related to aroma, crust color, crumb color, flavor and texture were used (vanilla smell, fruity smell, fermented smell, pale, brown, cream, yellow, sweet, fruity, bittersweet, spongy, moist, greasy, soft, fibrous), which were previously identified with experienced professional personnel. Likewise, using a 9-point hedonic scale (1 = I dislike it, 5 = I neither like it nor dislike it, 9 = I like it), panelists were asked to indicate their acceptability per sample and purchase intention with a 5-point scale (1 = I would not buy it, 5 = Yes, I would buy it) [22]. The samples were presented monadically, and table water was used to rinse the palate between each sample. In addition, the discriminative sorting task test was applied to 92 frequent consumers of panettone (60% women and 40% men). The predominant age range among consumers was 18 to 46 years old. Consumers received a tray with 6 disposable plates containing panettone samples in random order. They were asked to taste, smell and classify them based on the similarities and/or differences between them. To help the panelists, a form was provided so that they could make their notes; these presented clear and precise instructions to avoid errors. The general characteristics of the evaluation were explained to consumers. This was carried out in a single session divided into two stages [38]. In the first stage, consumers tasted the samples and in the second, they had to describe, using 4 or 5 words, the previously formed groups. Before performing the sensory analysis, all participants signed the informed consent letter, authorized by Certificate of Approval No. 003-2023-UNAB/CEPI, dated 22 November 2023, issued by the Research Ethics Committee of the National University of Barranca.

### 2.7. Statistical Analysis

A completely randomized design (CRD) was applied, with panettone brands as a factor and physicochemical analysis, color and texture as a response variable. For the sensory analysis, a completely randomized block design was used; for acceptability and purchase intention, the Friedman test was applied to the preference test. In the RATA method, an analysis of RATA descriptors and correspondence analysis were performed, and for the sorting task test, DISTATIS and FAST analysis were applied. The results are expressed as mean values ± standard deviation (SD) or frequencies depending on the type of data. In the case of finding significance, the Tukey comparison of means was carried out with 95% confidence. The data were processed using XLSTAT software version 2023, R Project and R Studio using the FactoMineR, SensoMineR and DistatisR package.

## 3. Results and Discussion

### 3.1. Chemical Composition

Table 2 presents statistically significant differences (*p* < 0.05) in all evaluated physicochemical properties among the different panettones. Regarding diameter, variations were observed among the samples, which clustered into two groups: the first group, composed of samples T3 and T6, showed statistically similar diameters, while the second group, consisting of the remaining samples (T1, T2, T4 and T5), displayed different diameters. The addition of leavening agents such as yeast or baking powder contributes to greater expansion during baking, resulting in a larger diameter of the product [39]. Likewise, fermentation time and conditions can affect the structure and texture of the dough, influencing the final product’s size [40]. A longer fermentation process or a higher temperature can favor greater dough expansion and, consequently, a larger diameter of the product [41].

Regarding weight, samples T1 and T2 recorded the highest weights, while T3 exhibited the lowest weight. These variations stem from the quantity and type of ingredients used in the dough, which can influence the final product’s weight [39]. Additionally, mixing, kneading and baking conditions affect the density and consistency of the dough, which in turn reflects in the panettone’s weight. A more uniform and controlled production process can lead to more consistent products in terms of weight. It is noteworthy that price can also influence weight, with more expensive products generally being heavier [42].

The moisture and ash content of all samples were within the maximum limits established by Peruvian legislation [43]. Sample T6 exhibited the highest moisture content, while T1, T2 and T3 showed lower values. The quantity of liquids (water, milk, eggs) and the presence of hydrating ingredients such as honey, sugar or molasses can generate variations in the product’s final moisture [42]. Regarding ash content, T3, T4 and T6 recorded the highest contents compared to the rest of the samples. The diversity of ingredients used, such as flour, yeast, fats and nuts, can influence the mineral content and, therefore, the ash content of the panettone [39].

pH and acidity are inversely related. Sample T1 was the most acidic, presenting the lowest pH value, consistent with the use of acidity regulators declared on the product label. Conversely, T4 was the least acidic sample. All acidity values (expressed as lactic acid) were within the maximum limits established by Peruvian sanitary regulations [43]. The addition of acidic ingredients such as dried fruits or natural yeast can increase acidity, while the use of sodium bicarbonate can influence pH [41].

Regarding fat content, commercial panettone exhibited higher fat content compared to the traditional one. Sample T1 had the highest fat content, followed by T2 and T3, while T4, T5 and T6 showed the lowest contents, mainly corresponding to traditional panettones. The quantity and type of fats added to the panettone dough may vary among different recipes and commercial brands. The use of butter, vegetable oil, margarine or other types of fats can significantly affect the final product’s fat content. Traditional panettones often have different fat quantities compared to commercial versions, which may also contain additives to extend their shelf life or improve their texture [39,42].

### 3.2. Instrumental Texture Analysis

Table 3 shows statistically significant differences (*p* < 0.05) in the texture properties of panettone, including hardness, elasticity, cohesiveness, chewiness and resilience. Regarding hardness, commercial panettones were notably firmer compared to traditional ones. This is attributed to commercial panettones containing a higher proportion of food additives, such as dough improvers, preservatives, or stabilizers, which affect the structure and texture of the product [44]. The production methods used for commercial and traditional panettones also vary in terms of mixing time, fermentation time and temperature and baking techniques. These factors can influence the formation of the crumb structure and crust of the panettone, affecting its final texture [36].

Elasticity and cohesiveness were similar among most samples (*p* > 0.05), except for T3 and T4, respectively. The elasticity of panettone dough is influenced by the amount of gluten present in the flour, as well as the addition of ingredients such as eggs, milk, or fats [30]. Prolonged kneading or longer fermentation time can cause the development of a stronger gluten network, resulting in a more elastic dough [45]. Some additives are also designed to strengthen gluten and improve dough elasticity, which can affect the texture of the final product. Cohesiveness refers to the ability of panettone dough to hold together and resist disintegration during chewing. Differences in cohesiveness between commercial and traditional panettones may be influenced by various factors related to production processes and ingredients used, such as the gluten content in the flour [46].

Chewiness was higher in most samples, except for T5 and T6, which exhibited lower values and were similar to each other (*p* > 0.05). Flours with high gluten content tend to produce more elastic products with better chewiness, while flours with low gluten content may result in more brittle or less chewy products [47]. The proper development of the gluten network during kneading and fermentation can enhance the chewiness of the panettone. Additionally, an overly dry panettone tends to be more difficult to chew, while one that is too moist may have an unpleasant mouthfeel [36].

Resilience, which refers to the product’s ability to recover after being deformed, was higher for T1, T2 and T5 (these samples were similar to each other, *p* > 0.05) and lower for the rest of the samples. The amount of gluten present in the flour and other ingredients such as fats, eggs and sugars can affect the structure and resilience of the product [Valcarcel, 2013]. The use of additives can strengthen the dough structure and improve its recovery capacity after deformation [48].

The texture values recorded for the studied panettones are lower than those found in similar research on panettone in terms of hardness and elasticity but very close in terms of cohesiveness, chewiness and resilience [36].

In commercial products, the use of enzymes is likely to considerably enhance cohesion, texture and therefore product quality [49]. Furthermore, the cohesiveness of panettones compared to bread is much higher, due to the configuration of the porous microstructures present in panettones [50]. Even in commercial products, the use of enzymes is likely to considerably improve the cohesiveness, texture and therefore the quality of the product [49].

### 3.3. Colorimetric Parameters

Table 4 indicates statistically significant differences (*p* < 0.05) in all evaluated colorimetric parameters, both in the crust and crumb of the panettone. Crust color serves as an indicator of the intensity of panettone baking. The crust exhibits lower L* values, indicating a higher propensity for browning due to Maillard reactions occurring during baking at high temperatures. These reactions result from the direct interaction of sugars with amino acids such as lysine, arginine and cysteine [51]. Additionally, the presence of saturated fatty acids also influences the Maillard reaction [52]. Sample T5 had the highest L* value, although statistically similar to T2, T4 and T6, while sample T1 recorded the lowest value, meaning darker. The baking conditions, such as temperature and time, could have affected color development [53]. Gentler baking or shorter baking times might have prevented excessive golden or toasted color formation, resulting in a higher L* value. The a* value presents positive values trending towards brown, with T1 and T3 being the most different samples. The use of sugars or honey can darken the color of the final product, while food colorings can alter the red or green tones [36,54]. Longer baking times or higher temperatures can result in increased sugar caramelization, affecting the a* parameter [55]. The b* value shows positive values trending towards yellow-brown, with significant differences between samples, T5 being the highest. Natural ingredients such as fruits, nuts, spices or food colorings can generate variation in the crust color of the panettone. Additionally, lipid oxidation and the degradation of natural pigments can cause changes in hue, saturation and color tone [56].

Regarding crumb color, the L* parameter was higher for T3, followed by T2 and T5, and lower values for T1, T4 and T6. These variations may arise from the moisture content of the panettones, which influences water absorption during dough preparation and the baking process, which in turn can affect color development in the crumb [30]. Additionally, the use of substitute flours, such as whole wheat, rye, spelt or blends of alternative flours, contains pigments that generate variations in luminosity [39]. The interaction of these flours with sugars, fats and egg yolks also influences crumb structure and its ability to reflect light, affecting the L* value. The a* value of the crumb shows significant differences, with T1 being higher than T3 and T4, while the rest of the samples were similar to each other. The addition of eggs provides a more yellow hue to the crumb, while sugar can caramelize during baking, affecting the final color [36]. The use of food colorings or additives can also enhance crumb color, affecting the tone. The b* and C* parameters exhibited a similar behavior, with T5 being the sample different from the rest. Positive values indicate the yellowish hue of the crumb. The hue (h*) was higher for T3 and T4 but lower in T1, T2, T5 and T6. The type of flour and fiber content generate color variations [46]. Whole wheat flours tend to produce darker crumbs due to their bran content, while refined flours can produce lighter crumbs. Higher fiber content can influence crumb texture and porosity, affecting light reflectance and thus the b* and C* values [40]. Additionally, the interaction between sugars and proteins can affect the Maillard reaction during baking, influencing crumb color and saturation [55]. In general, lower “a*” values and higher “b*” values indicate a predominance of the yellow tone, distinctive characteristics of panettone, even in some products where substitute flours are used [2].

**Figure 1:**
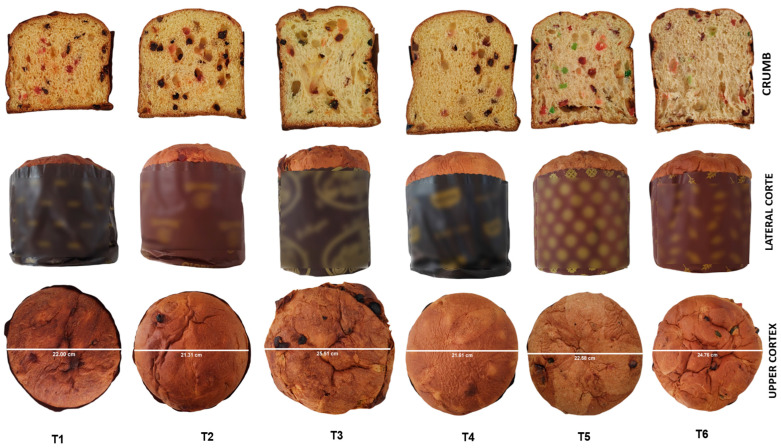
Images of the crumb, lateral crust and upper crust of commercial and traditional panettone samples. (T1: commercial panettone stuffed with raisins and candied fruits; T2: commercial panettone with raisins and candied fruit, free of potassium bromate; T3: premium commercial panettone; T4: commercial panettone with raisins and candied fruit; T5: traditional panettone with Andean ingredients of great nutritional value; T6: traditional panettone with Andean ingredients of great nutritional value with sweetener).

Figure 1 shows the cross sections of the panettone, showing the sponginess of each sample. T1, T2, T3 and T4 have slightly smaller alveoli, while T5 and T6 show larger pores, which are related to hardness. The lateral section shows the shape and size of the samples, with sample T1 being the lowest and most compact, possibly generated by the kneading and fermentation process, which affects the structure and elasticity of the dough. Inadequate kneading or insufficient fermentation can result in a denser and more compact panettone. The rest of the samples have a conical shape above the pyrotins. These differences can be attributed to the amount of dough placed in each pyrotin, an excessive amount of dough will probably form a conical protuberance at the top during the fermentation and baking process. During baking, the panettone dough expands due to fermentation and the release of gases. If the dough has a higher concentration of yeast or a longer rising time is provided, a conical bulge is more likely to form on the top of the panettone due to dough expansion. The exterior shape of the panettone is rough, showing very heterogeneous indentations or seams in all the samples, although T4 presented a more homogeneous surface; however, traces of burnt raisins were observed in some of them.

### 3.4. Sensory Analysis

#### 3.4.1. Sorting Test

**Figure 2:**
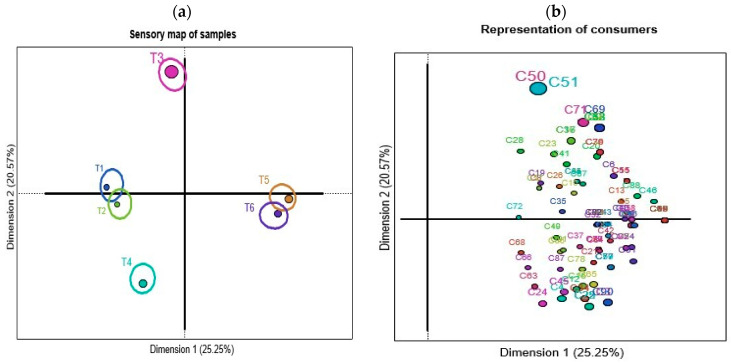

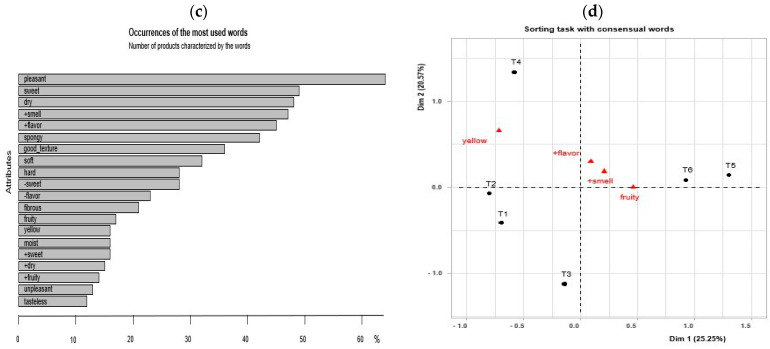
Sorting task graphs: (**a**) List of attributes or consumer descriptors, (**b**) dimension graph, (**c**) correspondence analysis (CA) and (**d**) correspondence relationship. (T1: commercial panettone stuffed with raisins and candied fruits; T2: commercial panettone with raisins and candied fruit, free of potassium bromate; T3: premium commercial panettone; T4: commercial panettone with raisins and candied fruit; T5: traditional panettone with Andean ingredients of great nutritional value; T6: traditional panettone with Andean ingredients of great nutritional value with sweetener).

Figure 2 shows the representation of panettone samples, consumers, generated attributes and the analysis of agreed words through the sorting task.

In Figure 2a, the map of panettone samples is observed, where this discriminative test generates four groups, where T1 and T2 form a group, T5 and T6 another group and samples T3 and T4 form independent groups; these last samples show a clear separation from the rest of the samples. Regarding the representation of consumers, an expected heterogeneity is revealed in the grouping of the samples, which can be explained by the interindividual variability between consumers. Figure 2c shows the list of terms or words most frequently generated by consumers when describing the samples, which are pleasant, sweet, dry, lots of smell, lots of flavor, spongy, good texture and soft, which recorded frequencies greater than 30% mention. Figure 2d presents the sensory map through CA explaining 45.82% of the total variability in the first two dimensions. The location of the samples is explained mainly by the type of products; the traditional samples are grouped by their ingredients and the commercial ones by certain characteristics reported by consumers. When observing the classification, samples T1 and T2 were described by a percentage of consumers as yellow in color; a similar behavior is observed for T4 characterized as yellow in color by another group of consumers, although this is different in other attributes from the rest of the samples. Sample T5 and T6 are characterized by having a lot of smell, flavor and fruits. Sample T3 is different from the other samples described with little yellow and low flavor, because it has a simpler formulation compared to the other samples. It uses basic ingredients such as wheat flour, sugar, vegetable fat, yeast, candied fruits, eggs and certified essences. Through the consensus of words, the most used attributes were yellow color, fruit, strong aroma and flavor. Sensorially, it is not expected that samples T5 and T6 were described in a similar way, since T6 has a sweetener that replaces sugar; however, consumers did not perceive this change, which indicates that it is viable to replace the sugar content in panettone. This method allowed us to record how consumers perceive the sensory attributes of panettone, reaching a consensus, grouping the samples according to their similarities. These dissimilarities or similarities can be generated by the quality of ingredients such as flour, yeast, dried fruit and spices, which can significantly affect the flavor and texture of the panettone [57,58].

#### 3.4.2. RATA (Rate-All-That-Apply)

**Figure 3:**
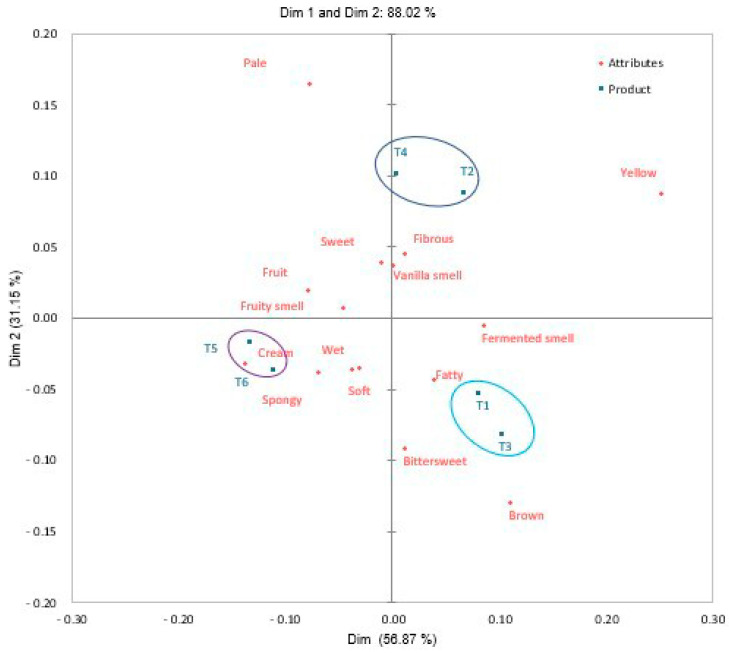
Sensory map obtained using the RATA technique. (T1: commercial panettone stuffed with raisins and candied fruits; T2: commercial panettone with raisins and candied fruit, free of potassium bromate; T3: premium commercial panettone; T4: commercial panettone with raisins and candied fruit; T5: traditional panettone with Andean ingredients of great nutritional value; T6: traditional panettone with Andean ingredients of great nutritional value with sweetener).

Figure 3 shows the sensory map obtained through the multivariate correspondence analysis of the RATA test. Using two dimensions, 88.02% of the total variability of the data was explained. The formation of three groups was observed, the first group was formed by T1 and T3 described as greasy, brown, fermented smelling and bittersweet. The second group was made up of T2 and T4 characterized by fibrous, sweet, vanilla and fruity smell. The third group was composed of T5 and T6 which are characterized as creamy, spongy, soft and moist.

Table 5 presents the sensory attributes evaluated using ANOVA for a univariate analysis, where significant differences (*p* < 0.05) are observed in all the attributes studied except for fibrous (*p* > 0.05). Regarding sample T3, I present lower values for sweet, vanilla smell, fruity smell and the presence of fruit, although I register higher values in the bittersweet flavor attribute. Sample T1 was described as the fattiest product, due to the presence of several high-fat ingredients and added fats in its formulation. T4 had higher values in the pale attributes, and T3 had significantly higher values in the brown color. Sample T4 indicates the use of “candied fruit” and dyes such as chlorophylls and carmines, which can add color to the final product. Sample T3 has fewer ingredients that can give a more intense and vibrant tone to the panettone, resulting in a brown color. Samples T5 and T6 presented lower values in the color yellow; however, they obtained higher significant values for the attributes fruit, creamy, spongy, soft and fruity smell; these last two attributes had similar values in sample T1.

The RATA method clearly shows that it is possible to discriminate products with similar sensory characteristics between commercial and traditional products. Sensory attributes may be perceived differently due to the ingredients of the product, incorporation of additives or improvers, production process, variations in the kneading, fermenting or baking method and addition of nuts (almonds, raisins and dried cranberries) that produce variations in the sensory profile (texture, aroma, flavor) of the panettone, affecting its level of liking [59,60,61].

#### 3.4.3. Liking and Purchase Intention

**Figure 4:**
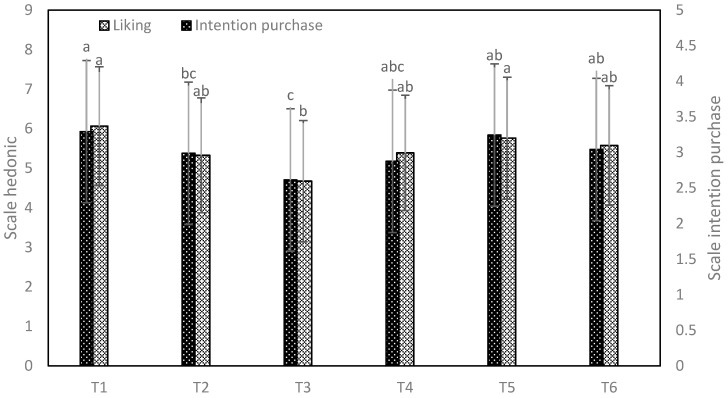
Acceptability and purchase intention graphs. (T1: commercial panettone stuffed with raisins and candied fruits; T2: commercial panettone with raisins and candied fruit, free of potassium bromate; T3: premium commercial panettone; T4: commercial panettone with raisins and candied fruit; T5: traditional panettone with Andean ingredients of great nutritional value; T6: traditional panettone with Andean ingredients of great nutritional value with sweetener). Different letters indicate significant differences.

Figure 4 shows the results of the liking and purchase intention of the different panettone, where significant differences were found (*p* < 0.05). Regarding liking, all samples were rated as moderately liked except for sample T3, which had lower values (I neither like nor dislike).

Sample T3 has a simpler formulation compared to other samples. The absence of aromatic ingredients such as candied fruits, nuts, spices or essences can result in a panettone with a flatter and less complex flavor profile. Therefore, a lower amount of these ingredients could negatively affect sensory acceptability. A similar behavior was observed in the commercial panettone (control), which presented lower acceptability compared to panettone made with pre-fermented doughs [30]. Purchase intention showed a similar behavior, the samples with the highest purchase intention were T1, T4, T5 and T6; a lower probability of purchase occurred for T2 and T3. A similar behavior was recorded [30] in traditional and pre-fermented panettone. Commercial panettones have great liking and purchase intention because they have a market of regular consumers, people who identify with the product and are familiar with certain characteristic sensory attributes of the panettone [62] indicates that the interaction of the eye-sight sense generates various taste sensations, specifically with consumers, which generate variations in the perception of flavor, smell and aroma, which directly influences their liking and purchase intention. Furthermore, market studies and consumer research can provide valuable information on consumer preferences and perceptions regarding commercial panettone.

## 4. Conclusions

This study focused on the evaluation of the sensory attributes of commercial and traditional panettone in the Peruvian market. Through RATA sensory tests, sorting task and physical–chemical, textural and colorimetric analysis, significant differences were detected between the different varieties of panettone. It was observed that commercial panettone has a higher fat content and greater hardness compared to traditional ones. The colorimetric parameters showed differences in both the crust and the crumb, with low values of luminosity in the crust indicating a greater tendency to darken, and high values of “b” in the crumb, which denotes a predominance of the yellow tone, characteristics distinctive of panettones. Sensory analysis using the sorting task test (discriminative) and RATA (descriptive) revealed the formation of four and three groups of samples, respectively. This indicates that, in the discriminative tests, consumers were able to distinguish between the different types of panettones, while in the descriptive tests, they grouped them according to similar characteristics. All the descriptive sensory attributes studied were differentiated by consumers, except for the “fibrous” attribute. Although acceptability (moderately like it) and purchase intention (would probably buy it) were similar for all samples, sample T3 had slightly lower values. Additional studies are recommended to evaluate the influence of ingredients, additives, production processes and variations in the kneading method on the nutritional and sensory profile of panettone.

## Figures and Tables

**Table 1:** Commercial and nutritional characteristics.

Sample	Description	Ingredients	Nutritional Information
T1	Panettone stuffed with raisins and candied fruits.	Fortified wheat flour (iron, vitamins: B1, B2, B3 and folic acid), water, sugar, raisins, candied fruit (papaya, sugar, orange peel, colorings (carmines: chlorophyllin, cupric complexes, potassium and sodium salts and curcumin), acidity regulator (citric acid), preservative substances (potassium sorbate), egg yolk, vegetable fat, anhydrous milk fat, ethyl alcohol, glucose syrup, wheat gluten, emulsifiers (monoglycerides and diglycerides of fatty acids, diacetyl tartaric and fatty acid esters of glycerol and sodium stearoyl lactylate), skimmed milk powder, flavorings, salt, yeast, preservatives (calcium propionate and ascorbic acid), colors (curcumin and annatto extract, norbixin base), flour treatment agent (L-ascorbic acid) and acidity regulator citric acid). May contain sesame, peanuts, tree nuts and soy.	Slice portion: 75 g. Per 100 g, it contains 379 kcal, fat 14.5 g, of which saturated fat 7.2 g, trans fat 0 g, carbohydrates 55.1 g, of which total sugars 20.5 g, proteins 7.0 g and sodium 129 mg.
T2	Panettone with raisins and candied fruit. Free of potassium bromate.	Fortified wheat flour (wheat, iron, vitamin B1, thiamin B2, vitamin B3 and folic acid), white sugar, water, raisins, candied fruit (papaya pulp, white sugar, preservatives, potassium sorbate (INS 202), acidity regulator: citric acid (INS 330), colors: carmine (INS 120) and chlorophyll (INS 141 (ii))); pasteurized egg yolk, anhydrous milk fat, margarine, natural yeast, ground candied orange peel, skimmed milk powder, emulsifiers: mono- and diglycerides of fatty acids of glycerol (INS 472 e), sodium carboxymethyl cellulose (INS 466), gum guar (INS 412) and calcium carbonate (INS 170 (i)), wheat gluten, salt, panettone flavoring, preservatives: calcium propionate (INS 282), emulsifier: sodium lactylate (INS 4811 (i)), colorants: curcumin (INS 100 (i)), annatto (INS 160 (ii)), flour treatment agents: alpha amylase from Aspergillus Oryza var. (INS 1100 (i)) and antioxidant: Tocopherols (INS 307b) (*, obtained from soybeans).	Serving size 80 g, serving per container 11. Energy (304 Kcal/ 1272 KJ). 100 g. Total fat 16 g, of which 8.6 g is saturated fat, trans fat 0 g, cholesterol (mg) 71, sodium (mg) 169, total carbohydrates (g) 52.6, of which dietary fiber 3.8 g, total sugars 17.4 g, added sugars 12 g, protein 6.4 g, calcium 82.8 mg, iron 4.5 mg.
T3	Premium panettone	Wheat flour, sugar, hydrogenated vegetable fat, natural yeast, candied fruit, eggs, raisins, soy lecithin, certified essences and colorings.	From 10 g of sample: Carbohydrates 57.2 g, energy 340.4 kcal, iron 3.2 mg, moisture 23.9 g, protein 9.9 g, ash 1.0 g, fiber 0.1 g, fat 8.0 g, calcium 73.0 mg.
T4	Panettone with raisins and candied fruit	Fortified wheat flour (iron, niacin, thiamine, riboflavin, folic acid), candied fruit (papaya pulp, invert syrup (refined sugar, glucose, acidity regulator (citric acid INS 330)), colors (chlorophylls, cupric complexes, potassium and sodium salts INS 141 (ii), carmines INS 120)), vegetable fat, sugar, raisins, natural yeast, pasteurized liquid egg yolk, wheat gluten, invert sugar syrup, glucose syrup, fresh yeast, emulsifiers (monoglycerides and diglycerides of fatty acids INS 471, diacetyl tartaric and fatty acid esters of glycerol INS 472e), modified potato starch, skimmed milk powder, modified starch maltodextrin, salt, artificial flavor, fructose, preservatives (ethyl alcohol, calcium propionate INS 282, sorbic acid INS 200), enzymes (xylanase, glucose oxidase), sweetener (sucralose INS 955), color (riboflavin INS 101(I)) and flour treatment agent (ascorbic acid SIN 300, alpha amylase from Aspergillus Oryza var. SIN 1100 (i). Contains gluten, egg and milk derivatives.	Approx. 15 servings per container. Serving size 1 slice (55 g). Energy value 190 kcal (795 KJ). Protein 3 g, carbohydrates (available) 32 g, of which sugars 12 g, dietary fiber 1 g, fat content 5 g, of which saturated fatty acids 2.5 g and trans fatty acids 0 g, sodium 125 mg. Nutrient Reference Value (NRV) percentages are based on a 2000 kcal (8370 KJ) diet.
T5	Panettone with Andean ingredients of great nutritional value.	Panettone wheat flour, water, dried cranberries, candied fruit, white sugar, margarine, egg yolks, pecans, gluten, honey, yeast, maltose, red quinoa, kiwicha, milk powder, improver, glucose, emulsifier, salt, panettone essence, vanilla essence, anti-mold and coloring.	Serving per container 8 g. Calories 320 Kcal, total carbohydrates 39 g, dietary fiber 10 g, sugars 22 g, total fat 10 g, saturated fat 5.1 g, trans fat 0 g, cholesterol 40 mg, sodium 122 mg, protein 16 g, vitamin E 20 mg, riboflavin 0.67 mg, thiamine 0.24 mg, niacin 5.3 mg, calcium 254 mg, iron 4.7 m.
T6	Panettone with Andean ingredients of great nutritional value with sweetener.	Panettone wheat flour, water, dried cranberries, candied fruit, Stevia sweetener, margarine, egg yolks, pecans, gluten, honey, yeast, maltose, red quinoa, kiwicha, milk powder, improver, glucose, emulsifier, salt, panettone essence, vanilla essence, anti-mold and coloring.	Serving per container 8 g. Calories 232 Kcal, total carbohydrates 39 g, dietary fiber 10 g, total fat 10 g, saturated fat 5.1 g, trans fat 0 g, cholesterol 40 mg, sodium 122 mg, protein 16 g, vitamin E 20 mg, riboflavin 0.67 mg, thiamine 0.24 mg, niacin 5.3 mg, calcium 254 mg, Iron 4.7 mg.

**Table 2:** Physicochemical characteristics of panettone in 100 g.

Sample	Diameter (cm)	Weight (g)	Moisture (%)	Ash (%)	pH	Acidity (% Lactic Acid)	Fat (%)
T1	22.017 ± 0.132 ^b^	930.300 ± 0.007 ^a^	20.419 ± 0.211 ^c^	0.568 ± 0.055 ^c^	4.550 ± 0.028 ^e^	0.409 ± 0.014 ^a^	17.457 ± 0.557 ^a^
T2	21.301 ± 0.303 ^b^	934.420 ± 0.035 ^a^	20.979 ± 0.497 ^c^	0.674 ± 0.021 ^bc^	4.785 ± 0.021 ^d^	0.310 ± 0.001 ^b^	13.596 ± 0.459 ^b^
T3	25.568 ± 0.613 ^a^	765.570 ± 0.007 ^c^	21.105 ± 0.325 ^c^	1.224 ± 0.191 ^a^	5.210 ± 0.014 ^b^	0.279 ± 0.005 ^b^	12.418 ± 0.377 ^b^
T4	21.596 ± 0.416 ^b^	863.670 ± 0.014 ^b^	22.807 ± 0.309 ^b^	0.933 ± 0.011 ^ab^	5.350 ± 0.028 ^a^	0.193 ± 0.003 ^c^	9.1183 ± 0.113 ^c^
T5	22.576 ± 0.213 ^b^	839.750 ± 12.710 ^b^	22.910 ± 0.233 ^b^	0.877 ± 0.031 ^bc^	5.005 ± 0.007 ^c^	0.318 ± 0.019 ^b^	9.7770 ± 0.149 ^c^
T6	24.785 ± 0.144 ^a^	838.190 ± 10.500 ^b^	25.458 ± 0.116 ^a^	0.959 ± 0.031 ^ab^	5.045 ± 0.035 ^c^	0.300 ± 0.008 ^b^	9.897 ± 0.022 ^c^

Values followed by different letters, in the same column, present significant differences (*p* ≤ 0.05) according to the Tukey test. (T1: commercial panettone stuffed with raisins and candied fruits; T2: commercial panettone with raisins and candied fruit, free of potassium bromate; T3: premium commercial panettone; T4: commercial panettone with raisins and candied fruit; T5: traditional panettone with Andean ingredients of great nutritional value; T6: traditional panettone with Andean ingredients of great nutritional value with sweetener).

**Table 3:** Instrumental texture profile (TPA) of the panettones.

Sample	Hardness (N)	Springiness	Cohesiveness	Chewiness (N)	Resilience
T1	1.259 ± 0.159 ^ab^	0.735 ± 0.034 ^a^	0.618 ± 0.068 ^a^	0.570 ± 0.112 ^a^	0.215 ± 0.024 ^a^
T2	1.148 ± 0.154 ^bc^	0.700 ± 0.036 ^ab^	0.603 ± 0.021 ^a^	0.485 ± 0.081 ^ab^	0.218 ± 0.015 ^a^
T3	1.198 ± 0.090 ^ab^	0.618 ± 0.021 ^b^	0.568 ± 0.044 ^a^	0.420 ± 0.066 ^ab^	0.175 ± 0.017 ^bc^
T4	1.500 ± 0.118 ^a^	0.680 ± 0.075 ^ab^	0.445 ± 0.010 ^b^	0.453 ± 0.061 ^ab^	0.165 ± 0.013 ^c^
T5	0.858 ± 0.085 ^c^	0.668 ± 0.048 ^ab^	0.543 ± 0.029 ^a^	0.315 ± 0.058 ^b^	0.208 ± 0.022 ^ab^
T6	1.002 ± 0.202 ^bc^	0.665 ± 0.027 ^ab^	0.568 ± 0.026 ^a^	0.375 ± 0.074 ^b^	0.198 ± 0.005 ^abc^

Values followed by different letters, in the same column, present significant differences (*p* ≤ 0.05) according to the Tukey test. (T1: commercial panettone stuffed with raisins and candied fruits; T2: commercial panettone with raisins and candied fruit, free of potassium bromate; T3: premium commercial panettone; T4: commercial panettone with raisins and candied fruit; T5: traditional panettone with Andean ingredients of great nutritional value; T6: traditional panettone with Andean ingredients of great nutritional value with sweetener).

**Table 4:** The color of the crumb and crust of the panettone.

Sample	Crust					
	L*	a*	b*	C*	h*	Color Simulation
T1	41.740 ± 1.870 ^c^	10.250 ± 0.599 ^c^	12.260 ± 2.050 ^d^	16.000 ± 1.940 ^d^	49.830 ± 3.350 ^c^	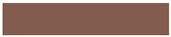
T2	47.293 ± 1.395 ^abc^	12.730 ± 0.606 ^ab^	17.930 ± 2.060 ^bc^	22.000 ± 2.020 ^bc^	54.510 ± 1.800 ^abc^	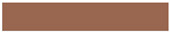
T3	43.657 ± 1.419 ^bc^	11.687 ± 0.931 ^bc^	15.970 ± 2.120 ^cd^	19.790 ± 2.260 ^cd^	53.693 ± 1.553 ^bc^	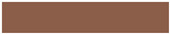
T4	49.967 ± 1.174 ^ab^	13.567 ± 0.186 ^a^	21.263 ± 0.8490 ^ab^	25.220 ± 0.815 ^ab^	57.453 ± 0.669 ^ab^	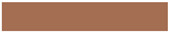
T5	52.860 ± 4.660 ^a^	14.020 ± 0.370 ^a^	23.687 ± 1.635 ^a^	27.533 ± 1.478 ^a^	59.327 ± 1.675 ^a^	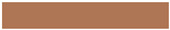
T6	47.140 ± 2.150 ^abc^	13.227 ± 0.415 ^ab^	19.117 ± 0.731 ^abc^	23.247 ± 0.583 ^abc^	55.303 ± 1.492 ^ab^	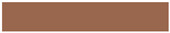
	Crumb					
T1	64.360 ± 3.370 ^b^	4.717 ± 0.635 ^a^	26.320 ± 2.130 ^a^	26.740 ± 2.200 ^a^	79.870 ± 0.639 ^b^	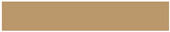
T2	67.493 ± 1.118 ^ab^	3.353 ± 0.690 ^ab^	26.730 ± 0.723 ^a^	26.943 ± 0.780 ^a^	82.867 ± 1.355 ^ab^	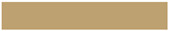
T3	72.457 ± 1.217 ^a^	2.277 ± 0.515 ^b^	26.180 ± 4.480 ^a^	26.280 ± 4.490 ^a^	85.043 ± 0.657 ^a^	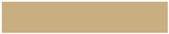
T4	65.290 ± 2.330 ^b^	2.103 ± 0.512 ^b^	24.817 ± 1.383 ^ab^	25.210 ± 1.218 ^ab^	85.193 ± 0.908 ^a^	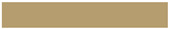
T5	67.887 ± 0.687 ^ab^	3.273 ± 0.624 ^ab^	18.637 ± 1.374 ^b^	18.937 ± 1.240 ^b^	79.920 ± 2.630 ^b^	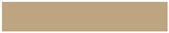
T6	66.030 ± 1.890 ^b^	3.420 ± 0.406 ^ab^	20.590 ± 1.850 ^ab^	20.870 ± 1.890 ^ab^	80.577 ± 0.484 ^b^	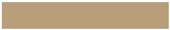

Values followed by different letters, in the same column, present significant differences (*p* ≤ 0.05) according to the Tukey test. (T1: commercial panettone stuffed with raisins and candied fruits; T2: commercial panettone with raisins and candied fruit, free of potassium bromate; T3: premium commercial panettone; T4: commercial panettone with raisins and candied fruit; T5: traditional panettone with Andean ingredients of great nutritional value; T6: traditional panettone with Andean ingredients of great nutritional value with sweetener).

**Table 5:** Analysis of RATA descriptors.

Sample	T1	T2	T3	T4	T5	T6
Vanilla Smell	2.268 ± 1.669 ^a^	2.244 ± 1.625 ^a^	1.798 ± 1.554 ^b^	2.107 ± 1.540 ^ab^	2.137 ± 1.663 ^ab^	2.125 ± 1.575 ^ab^
Fruity Smell	2.482 ± 1.660 ^a^	2.387 ± 1.547 ^ab^	2.018 ± 1.603 ^b^	2.214 ± 1.646 ^ab^	2.518 ± 1.656 ^a^	2.589 ± 1.572 ^a^
Fermented Smell	2.006 ± 1.697 ^abc^	1.702 ± 1.569 ^bc^	2.161 ± 1.643 ^a^	2.095 ± 1.806 ^ab^	1.685 ± 1.583 ^bc^	1.673 ± 1.576 ^c^
Pale	1.583 ± 1.538 ^c^	2.220 ± 1.643 ^ab^	1.518 ± 1.548 ^c^	2.548 ± 1.655 ^a^	2.250 ± 1.684 ^ab^	2.036 ± 1.695 ^b^
Brown	2.946 ± 1.653 ^a^	2.185 ± 1.733 ^b^	2.970 ± 1.852 ^a^	1.839 ± 1.714 ^b^	2.208 ± 1.730 ^b^	2.232 ± 1.608 ^b^
Cream	2.125 ± 1.772 ^b^	2.042 ± 1.721 ^b^	2.119 ± 1.702 ^b^	2.185 ± 1.705 ^b^	3.006 ± 1.502 ^a^	2.780 ± 1.506 ^a^
Yellow	2.631 ± 1.749 ^a^	2.720 ± 1.667 ^a^	2.399 ± 1.638 ^a^	2.399 ± 1.692 ^a^	1.381 ± 1.593 ^b^	1.452 ± 1.496 ^b^
Sweet	2.673 ± 1.658 ^a^	2.419 ± 1.691 ^a^	1.935 ± 1.657 ^b^	2.446 ± 1.673 ^a^	2.470 ± 1.695 ^a^	2.363 ± 1.746 ^ab^
Fruit	2.083 ± 1.752 ^ab^	2.095 ± 1.653 ^ab^	1.940 ± 1.719 ^b^	2.298 ± 1.683 ^ab^	2.506 ± 1.744 ^a^	2.482 ± 1.764 ^a^
Bittersweet	1.798 ± 1.669 ^ab^	1.506 ± 1.472 ^b^	2.006 ± 1.636 ^a^	1.485 ± 1.536 ^b^	1.827 ± 1.734 ^ab^	1.762 ± 1.696 ^ab^
Spongy	2.524 ± 1.795 ^ab^	2.065 ± 1.692 ^c^	2.196 ± 1.664 ^bc^	2.250 ± 1.662 ^bc^	2.756 ± 1.697 ^a^	2.732 ± 1.746 ^a^
Wet	1.958 ± 1.793 ^ab^	1.607 ± 1.586 ^b^	1.690 ± 1.484 ^ab^	1.732 ± 1.514 ^ab^	1.881 ± 1.666 ^ab^	2.024 ± 1.713 ^a^
Fatty	1.988 ± 1.720 ^a^	1.536 ± 1.492 ^b^	1.631 ± 1.534 ^b^	1.536 ± 1.464 ^b^	1.560 ± 1.535 ^b^	1.708 ± 1.624 ^ab^
Soft	2.292 ± 1.745 ^a^	1.804 ± 1.693 ^b^	1.970 ± 1.658 ^ab^	2.060 ± 1.722 ^ab^	2.238 ± 1.765 ^a^	2.274 ± 1.891 ^a^
Fibrous	2.095 ± 1.745 ^a^	2.470 ± 1.663 ^a^	2.196 ± 1.693 ^a^	2.268 ± 1.676 ^a^	2.280 ± 1.764 ^a^	2.179 ± 1.721 ^a^

Values followed by different letters, in the same column, present significant differences (*p* ≤ 0.05) according to the Tukey test. (T1: commercial panettone stuffed with raisins and candied fruits; T2: commercial panettone with raisins and candied fruit, free of potassium bromate; T3: premium commercial panettone; T4: commercial panettone with raisins and candied fruit; T5: traditional panettone with Andean ingredients of great nutritional value; T6: traditional panettone with Andean ingredients of great nutritional value with sweetener).

## Data Availability

The original contributions presented in the study are included in the article, further inquiries can be directed to the corresponding author.
